# Ca^2+^-Dependent Transcriptional Repressors KCNIP and Regulation of Prognosis Genes in Glioblastoma

**DOI:** 10.3389/fnmol.2018.00472

**Published:** 2018-12-18

**Authors:** Isabelle Néant, Jacques Haiech, Marie-Claude Kilhoffer, Francisco J. Aulestia, Marc Moreau, Catherine Leclerc

**Affiliations:** ^1^Centre de Biologie du Développement (CBD), Centre de Biologie Intégrative (CBI), CNRS, UPS, Université de Toulouse, Toulouse, France; ^2^Laboratoire d’Excellence Medalis, CNRS, LIT UMR 7200, Université de Strasbourg, Strasbourg, France; ^3^Department of Basic Science and Craniofacial Biology, NYU College of Dentistry, New York, NY, United States

**Keywords:** Ca^2+^ signaling, neuronal Ca^2+^ sensors, KCNIP, glioblastoma multiform, cancer stem cells (CSC), quiescence

## Abstract

Glioblastomas (GBMs) are the most aggressive and lethal primary astrocytic tumors in adults, with very poor prognosis. Recurrence in GBM is attributed to glioblastoma stem-like cells (GSLCs). The behavior of the tumor, including proliferation, progression, invasion, and significant resistance to therapies, is a consequence of the self-renewing properties of the GSLCs, and their high resistance to chemotherapies have been attributed to their capacity to enter quiescence. Thus, targeting GSLCs may constitute one of the possible therapeutic challenges to significantly improve anti-cancer treatment regimens for GBM. Ca^2+^ signaling is an important regulator of tumorigenesis in GBM, and the transition from proliferation to quiescence involves the modification of the kinetics of Ca^2+^ influx through store-operated channels due to an increased capacity of the mitochondria of quiescent GSLC to capture Ca^2+^. Therefore, the identification of new therapeutic targets requires the analysis of the calcium-regulated elements at transcriptional levels. In this review, we focus onto the direct regulation of gene expression by KCNIP proteins (KCNIP1–4). These proteins constitute the class E of Ca^2+^ sensor family with four EF-hand Ca^2+^-binding motifs and control gene transcription directly by binding, *via* a Ca^2+^-dependent mechanism, to specific DNA sites on target genes, called downstream regulatory element (DRE). The presence of putative DRE sites on genes associated with unfavorable outcome for GBM patients suggests that KCNIP proteins may contribute to the alteration of the expression of these prognosis genes. Indeed, in GBM, *KCNIP2* expression appears to be significantly linked to the overall survival of patients. In this review, we summarize the current knowledge regarding the quiescent GSLCs with respect to Ca^2+^ signaling and discuss how Ca^2+^ *via* KCNIP proteins may affect prognosis genes expression in GBM. This original mechanism may constitute the basis of the development of new therapeutic strategies.

## Introduction

Among tumors of the central nervous system, glioblastomas (GBMs) are the most aggressive and lethal primary astrocytic tumors in adults, with very poor prognosis (Louis et al., 2016; Lapointe et al., 2018). More than 90% of the patients show recurrence after therapies combining surgical resection, radiotherapy, and temozolomide (TMZ)-based chemotherapy, and the mean survival period rarely exceeds 2 years (Stupp et al., 2005). According to the cancer stem cell model, recurrence in GBM is attributed to a small sub-population of tumor cells called glioblastoma stem-like cells (GSLCs). These GSLCs have stem-like properties and are responsible for the initiation and the growth of the tumors (Visvader and Lindeman, 2008). Indeed, the GSLCs provide all the subtypes of cells that comprise the tumor including some pseudo-endothelial cells (Ricci-Vitiani et al., 2010). GSLCs are characterized by a molecular signature which combines markers of neural and/or embryonic stem cells and of mesenchymal cells. Numerous studies support the proposal that the behavior of the tumor, including proliferation, progression, invasion, and significant resistance to therapies, is determined by the self-renewing properties of the GSLCs (Stupp et al., 2005; Bao et al., 2006; Hegi et al., 2006; Stupp and Hegi, 2007; Murat et al., 2008). More importantly, this high resistance capacity to TMZ treatment have been attributed to slow cycling or relatively quiescent GSLCs (Pistollato et al., 2010; Deleyrolle et al., 2011). Quiescent GSLCs have been identified *in vivo* in a mouse model of GBM (Chen et al., 2012) and in human GBM tumors (Ishii et al., 2016). Thus, targeting GSLCs and their stem cell-like properties may constitute one of the possible therapeutic challenges to significantly improve anti-cancer treatment regimens for GBM.

Ca^2+^ is a crucial second messenger (Carafoli and Krebs, 2016) that controls a wide variety of cell functions from cell proliferation and apoptosis to organogenesis (Berridge et al., 2000; Machaca, 2011; Moreau et al., 2016). Thus, the intracellular Ca^2+^ concentration ([Ca^2+^]i) is tightly regulated and involves Ca^2+^ channels, pumps, and exchangers both at the plasma membrane and at the membrane of endoplasmic reticulum, mitochondria, or Golgi apparatus (Bootman, 2012; Humeau et al., 2018). In addition, changes in [Ca^2+^]i do not proceed in a stereotypical manner. The Ca^2+^ signal can be described by its amplitude (variations of [Ca^2+^]i levels) and by its spatial (sources of Ca^2+^; organelles where changes occur) and time-dependent (duration, frequency) components (Berridge, 1992; Haiech et al., 2011; Smedler and Uhlén, 2014; Monteith et al., 2017). The remodeling of Ca^2+^ signaling contributes also to cancer hallmarks such as excessive proliferation, survival, or resistance to cell death (Roderick and Cook, 2008; Prevarskaya et al., 2014) and accumulating evidence suggests that Ca^2+^ is also an important positive regulator of tumorigenesis in GBM (Robil et al., 2015; Leclerc et al., 2016). Interestingly, screening of the Prestwick Chemical library identified bisacodyl, an organic compound used as a stimulant laxative drug, with cytotoxic effect on quiescent GSLCs (Zeniou et al., 2015). Bisacodyl inhibits Ca^2+^ release from inositol 1,4,5-triphosphate-dependent Ca^2+^ stores without affecting the store-operated Ca^2+^ entry (SOCE) (Dong et al., 2017). These data exemplify the fact that Ca^2+^ channels, pumps, and exchangers may represent potential therapeutic targets. In this review, we will summarize the current knowledge regarding the quiescent GSLCs with respect to Ca^2+^ signaling and describe an original mechanism by which Ca^2+^ can activate some genes involved in the prognosis of GBM in order to propose new strategies to explore the molecular basis of GBM development for therapeutic issues.

## Transition from Proliferation to Quiescence and Ca^2+^ Signaling

Quiescent cells are non-proliferative cells, arrested in a specific phase of the cell cycle called G0 (Coller et al., 2006). Quiescence is not a prolonged G1 phase and in contrary to the cell-cycle arrest observed in differentiation or senescence, it is reversible. Transcriptional profiling data reveal that quiescent stem cells are characterized by a common set of genes which are either downregulated, these are genes associated with cell-cycle progression (i.e., *CCNA2*, *CCNB1*, and *CCNE2*), or upregulated and classified as tumor suppressors, including the cyclin-dependent kinase inhibitor p21 (*CDKN1A*) and the G0/G1 switch gene 2 (*G0S2*) (Yamada et al., 2012; Cheung and Rando, 2013). Quiescence represents a strategy for GSLCs to evade killing. It is thus vital to better characterize the quiescent GSLCs and to understand the mechanisms involved in the transition from a proliferative to a quiescence state. Quiescence is actively regulated by signals provided by the stem cell microenvironment. In GBM, quiescent cells are found close to necrotic tissues, in specific niches characterized by a hypoxic (Pistollato et al., 2010; Persano et al., 2011; Ishii et al., 2016) and acidic microenvironment (Garcia-Martin et al., 2006; Honasoge et al., 2014).

A recent study suggests that Ca^2+^ is an important regulator of the balance between quiescence and proliferation in hematopoietic stem cell (HSC) (Umemoto et al., 2018). In HSCs, re-entry into cell-cycle requires Ca^2+^ influx through Cav1 voltage-dependent Ca^2+^ channel and the resultant activation of mitochondria. Recent findings in our group showed that Ca^2+^ signaling is also required for GBM stem cells quiescence. On GSLCs lines, established from surgical resections of primary GBMs, we showed that change in Ca^2+^ homeostasis is an important actor of the transition from proliferation to quiescence. In order to analyze the signals underlying this switch, we modified the culture condition by lowering the extracellular pH from pH 7.5 to 6.5. GSLCs kept in such conditions for 5 days enter G0. This simple protocol allowed to reversibly maintain GSLCs in a proliferating or in quiescent state (Zeniou et al., 2015; Aulestia et al., 2018). A RNAseq analysis, focusing on the Ca^2+^ toolkit genes (Robil et al., 2015), established the transcriptional profiles of these proliferative and quiescent GSLCs and revealed that genes regulating plasma membrane Ca^2+^ channels (*CACNA2D1* and *ORAI2*) and mitochondrial Ca^2+^-uptake (*MCU*, *MICU1*, *MICU2*, and *VDAC1*) are downregulated in quiescence while others are upregulated (*CACNB1*, *CAPS*, and *SLC8B1*). A functional analysis through a bioluminescent Ca^2+^ imaging approach showed that quiescence in GSLCs does not involve Cav1 channels like in HSCs, but is rather due to the modification of the kinetics of the store-operated Ca^2+^ entry (SOCE), mediated by plasma membrane ORAI channels associated with the ER membrane protein STIM1. The inhibition of store-operated channels (SOC) by SKF96365 triggers quiescence, further supporting the crucial role of SOC in quiescence in GSLCs. Interestingly, the use of bioluminescent Ca^2+^ reporter targeted to mitochondria revealed that this change in SOCE kinetics is due to an increased capacity of quiescent GSLCs’ mitochondria to capture Ca^2+^ and not to the modification of the SOCE mechanism itself (Aulestia et al., 2018). These data highlight the importance of mitochondria as regulator of Ca^2+^ homeostasis.

Over the past decade, many studies have identified changes in the expression levels of proteins involved in Ca^2+^ homeostasis such as Ca^2+^ channels, pumps, and exchangers and established that some of these proteins contribute to tumorigenesis through regulation of proliferation, migration, or apoptosis (Monteith et al., 2012; Leclerc et al., 2016). As a second messenger, Ca^2+^ is also an important regulator of gene expression. This occurs either indirectly, *via* changes in the transactivating properties of transcription factors following the activation of Ca^2+^-dependent kinases and/or phosphatases (Dolmetsch, 2001; West et al., 2001; Kornhauser et al., 2002; Spotts et al., 2002), or directly *via* EF hand Ca^2+^-binding proteins which belongs to a group of four proteins (KCNIP1–4) (Mellström et al., 2008). The identification of new therapeutic targets now requires not only to target the identified proteins but also to analyze the molecular mechanisms responsible for the changes in gene expression observed in cancer cells. In this review, we choose to focus on the direct mode of action of Ca^2+^ on transcription with the implication of KCNIPs in GBM.

## The Family of Neuronal Ca^2+^ Sensors: KCNIPs

Potassium channel-interacting proteins (KCNIPs), which constitute the class E of Ca^2+^ sensor family, are globular proteins of 217–270 amino acids in size, with variable N- and C-termini and a conserved core domain containing four EF-hand Ca^2+^-binding motifs (EF-1, EF-2, EF-3, and EF-4). Among the four EF hands, EF-1 is not able to bind Ca^2+^ (Buxbaum et al., 1998; Carrión et al., 1999; An et al., 2000). *Drosophila melanogaster* has a single *Kcnip*, whereas mammals have four *KCNIP*s (*KCNIP*1–4) and several alternatively spliced variants (Burgoyne, 2007). In mammals, the four *KCNIP*s are predominantly expressed in adult brain, with specific or overlapping patterns according to the tissues (Rhodes, 2004; Xiong et al., 2004; Pruunsild and Timmusk, 2005). KCNIP3, also called calsenilin, KChIP3, and DREAM [i.e., Downstream Regulatory Element (DRE) Antagonist Modulator] is also found in the thyroid gland (Dandrea et al., 2005; Rivas et al., 2009) and in the hematopoietic progenitor cells (Sanz, 2001). KCNIP2 and KCNIP3 are found in T and B lymphocytes (Savignac et al., 2005, 2010). During mouse development, *Kcnip3* transcript first occurs at E10.5 (Spreafico et al., 2001) and *Kcnip1*, *2*, and *4* are not detected before E13 (Pruunsild and Timmusk, 2005). In the fish *Danio rerio*, the embryonic expressions of *kcnip1b* and *kcnip3* are not detectable before somitogenesis (Stetsyuk et al., 2007) and in the amphibian *Xenopus laevis* among the four *kcnip*s, only *kcnip1* is expressed at all developmental stages, from fertilized egg to the tadpole stages. By contrast, the transcripts for *kcnip2*, *kcnip3*, and *kcnip4* are expressed at later stages, after the specification of neural territories (Néant et al., 2015).

KCNIP proteins are known to control gene transcription directly by binding, *via* a Ca^2+^-dependent mechanism, to specific DNA sites, called DRE, of target genes. DRE sites are localized in the proximal 5′ sequence of the gene, downstream of the TATA box and upstream of the start codon, with the sequence GTCA forming the central core of the DRE site (Carrión et al., 1999; Ledo et al., 2000). This mechanism has been particularly well described for KCNIP3 (DREAM). When the intracellular Ca^2+^ level is low, KCNIP3 is bound as tetramer to the DRE sites, acting mainly as a transcriptional repressor. An increase in intracellular Ca^2+^ leads to dissociation of the KCNIP3 tetramer from its DRE site, thus allowing transcription (Carrión et al., 1999). KCNIP3 can affect transcription by acting either as a transcriptional repressor (Carrión et al., 1999; Link, 2004) or activator (Scsucova, 2005; Cebolla et al., 2008). In a more recent study, KCNIP3 has been shown to be required for human embryonic stem cells (hESCs) survival and to maintain hESCs pluripotency (Fontán-Lozano et al., 2016). KCNIP3 was initially the only Ca^2+^ sensor known to bind to DRE sites and to directly regulate transcription in a Ca^2+^-dependent manner (Mellström and Naranjo, 2001). However, all the four KCNIPs exhibit DRE-binding site affinity as homo or heterotetramers and act as Ca^2+^-dependent transcriptional regulators (Osawa et al., 2001; Craig et al., 2002; Link, 2004), allowing functional redundancy. KCNIP2 and KCNIP3 interactions are indirectly evidenced by two-hybrid and immunoprecipitation experiments (Savignac et al., 2005) and by the fact that KCNIP3 and KCNIP2 are both able to physically interact with EF-hand mutated KCNIP3 and that such associations still inhibit DRE-dependent gene expression (Gomez-Villafuertes, 2005; Savignac et al., 2005). *In vivo* studies also argue for the existence of compensatory mechanisms and the formation of functional KCNIP heterotetramers. Particularly, while in cortico-hippocampal neurons from *Kcnip3* knockdown mice, the expression levels of KCNIP3 target genes such as *Npas4* and *c-fos* are not significantly modified, the additional invalidation of *Kcnip2* with an antisense lentiviral vector (in this *Kcnip3* KO context) results in a significant increase in the expression of these KCNIP3-dependent target genes (Mellström et al., 2014). In amphibian embryos, we demonstrated that Kcnip1 binds DRE sites in a Ca^2+^-dependent manner. *Kcnip1* is the earliest *kcnip* gene expressed in *X. laevis* embryo. Its transcripts are timely and spatially present in the presumptive neural territories. In this *in vivo* model, loss of function experiments indicate that Kcnip1 is a Ca^2+^-dependent transcriptional repressor that controls the size of the neural plate by regulating the proliferation of neural progenitors (Néant et al., 2015).

## KCNIP Proteins in Glioblastoma

To the best of our knowledge, no published work has analyzed the expression of *KCNIP*s in GSLCs or more generally in cancer stem cells. Using the UALCAN server (Chandrashekar et al., 2017), it was possible to compare gene expression in normal brain tissues versus GBM multiform. *KCNIP1–4* are expressed in normal tissues at comparable levels. Interestingly, in GBM tissues while *KCNIP1* is significantly upregulated compared to its levels in normal brain tissues, *KCNIP2* and *KCNIP3* are strongly downregulated (Table 1). Although *KCNIP4* expression appears downregulated in GBM, the results are not statistically significant. This is probably due to large variability of *KCNIP4* expression in normal brain tissues and the small number of samples analyzed. In terms of survival, only *KCNIP2* expression is relevant. Among GBM patients, those with high *KCNIP2* expression appear to have a significant reduction in their overall survival time (UALCAN analysis). A recent study incidentally provides additional information on *KCNIP* expression in BT189 GSLC (Wang et al., 2018). Wang and coworkers analyzed the function of ING5, an epigenetic regulator overexpressed in GBM, and showed that ING5 promotes GSLCs self-renewal capabilities. Using the fluorescent Ca^2+^ probe fluo3, these authors showed that [Ca^2+^]i increases in cells overexpressing ING5. This increase in the resting Ca^2+^ level is required to maintain GSLCs’ self-renewal. Conversely, ING5 knockdown results in a strong reduction of the resting [Ca^2+^]i. To decipher further how ING5 is acting, they performed the transcriptomic analysis of GSLC cells where ING5 is knockdown. Among the differentially expressed genes, several Ca^2+^ channels were identified as upregulated by ING5, including some subunits of L and P/Q types of voltage-gated Ca^2+^ channels (*CACNA1F*, *1S*, *1D*, and *1C* and *CACN1A*, respectively) and of transient receptor potential cation channels (*TRPC3*, *C5*, *C4*, and *M1*). Of note, close examination of this list revealed that *KCNIP1–4* are indeed expressed in the BT189 GSLCs, although with different expression levels, and that *KCNIP2* is upregulated by ING5 in this GLSC (see Supplementary Table S1 in Wang et al., 2018).

**Table 1:** KCNIP genes expression in glioblastoma multiform.

Gene	Gene expression in normal brain tissues (*n* = 5) maximum – median – minimum	Gene expression in glioblastoma multiform (GBM) tissues (*n* = 156) maximum – median – minimum	Statistical significance at 0.05
*KCNIP1*	42.014 – **38.869** – 34.986	229.234 – **50.603** – 0.603	1.829 E-04
*KCNIP2*	85.765 – **68.456** – 16.25	23.856 – **6.02** – 0.222	4.978 E-02
*KCNIP3*	51.355 – **51.275** – 49.132	33.57 – **8.244** – 0.43	4.279 E-09
*KCNIP4*	77.262 – **53.813** – 10.084	25.877 – **4.346** – 0.501	7.312 E-02


*Gene expression is presented as a number of transcripts for each KCNIP genes per million of total transcripts. Data extracted using the UALCAN server (http://ualcan.path.uab.edu/index.html). Genes and proteins symbols are formatted according to the specific conventions particular to each organism (www.biosciencewriters.com).*

These data suggest a role of KCNIP proteins in stemness maintenance and dormant status of the GSLCs. The importance of KCNIPs in GBM is further emphasized by the presence of potential DRE sites within the proximal promoter of *MCU* and *MICU*2, two genes downregulated in quiescent GSLCs (Aulestia et al., 2018) and within the proximal promoters of *TRPC*5, *TRPC*4, and *TRPM*1, genes from the TRP family upregulated by the epigenetic factor ING5 in BT189 GSLC (Wang et al., 2018; Table 2).

**Table 2:** Candidate genes with putative DRE site and expressed in GSLCs.

Gene	Function	Expression in GSLCs	Reference	Number of putative DRE sites	Sense/position in bp^a^	Sequence in *Homo sapiens* (the core sequence is underlined)
*MCU*^b^	Mitochondrial calcium uniporter	Downregulated in the quiescent state	Aulestia et al., 2018	3	S/-240 AS/-207 S/-101	5′ tttgggt**gtcaa**ttatgggt 3′ 5′ cccgtaa**ttgac**tatgtccc 3′ 5′ caactca**gtcaa**gggcttta 3′
*MCUb*	Mitochondrial calcium uniporter beta subunit			2	AS/-70 AS/-36	5′ ccaggcgc**tgac**gaggagcc 3′ 5′ tgcgccgc**tgac**gcctgcgg 3′
*MICU1*	Mitochondrial calcium uptake 1			NF		
*MICU2*	Mitochondrial calcium uptake 2			1	AS/-199	5′ ggatggga**tgac**aggaagag 3′
*VDAC1*	Voltage-dependent anion channel 1			NF		
*TRPC3*	Transient receptor potential cation channel subfamily C member 3	Upregulated by ING5	Wang et al., 2018	NF		
*TRPC4*	Transient receptor potential cation channel subfamily C member 4			4	AS/-620 AS/-582 S/-355 S/-76	5′ ggctgga**tgac**ggctggctg 3′ 5′ cactggc**tgac**ctcaagcag 3′ 5′ atccgct**gtca**gccgtggga 3′ 5′ ccgcgcc**gtca**gtcctcgga 3′
*TRPC5*^b^	Transient receptor potential cation channel subfamily C member 5			4	S/-478 S/-465 S/-451 AS/-421	5′ cctacagt**gtca**gctacccc 3′ 5′ ctacccct**gtca**gtttcccc 3′ 5′ ttccccgt**gtca**gtttcttc 3′ 5′ attgtgtg**tgac**tggctgcg 3′
*TRPM1*	Transient receptor potential cation channel subfamily M member 1			1	S/-34	5′ ccgaggga**gtca**gcagggtg 3′


*^a^Position upstream from the ATG; S, sense; AS, anti-sense; ^*b*^putative DRE sites in close proximity. NF, not found, in accordance with the criteria in proximal 5′ upstream sequence between the tata box and the start codon (see details in text).*

## Regulation of GBM Prognosis Genes by KCNIP Proteins

**FIGURE 1:**
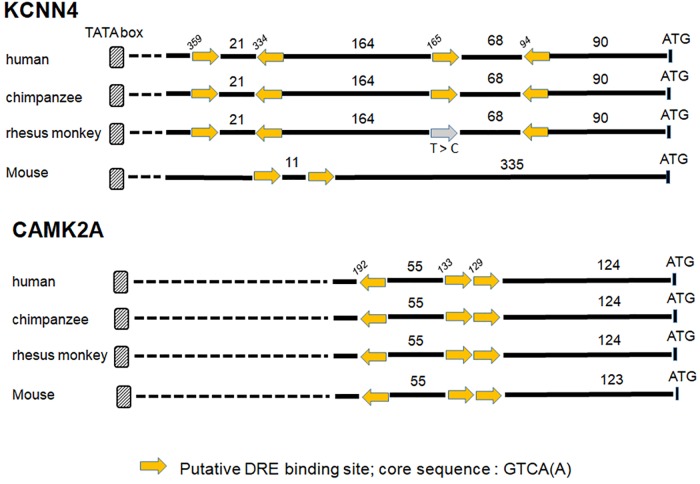
Putative DRE sites for two prognosis genes KCNN4 and CAMK2A. The proximal promoters of primates (human, chimpanzee, and rhesus monkey) and mouse are highly conserved for *KCNN4* and *CAMK2A* genes, the putative DRE-binding sites within these proximal promoters are positioned regarding to ATG (numbers in italic), the lengths of the 5′ fragment sequences are expressed in bp, and the yellow arrow gives the sequence orientation (see Table 3 for DRE sequence details). A point mutation (T to C) in a “sense” DRE site within rhesus monkey sequence is mentioned by the gray arrow.

Ion channels are now considered as important actors in cancers. Recent studies using microarray datasets of glioma samples obtained from the CGGA (Chinese Glioma Genome Atlas) and the TCGA (The Cancer Genome Atlas) identified genes belonging to the Ca^2+^ signaling machinery as new candidate genes that can predict GBM patients with high risk of unfavorable outcome (Wang et al., 2016; Zhang et al., 2017, 2018). These genes, listed in Table 3, are ion channels genes namely *CACNA1H*, a T-type Ca^2+^ channel (Cav3.2); *KCNN4*, a potassium Ca^2+^-activated channel (KCa3.1); *KCNB1*, a voltage-gated potassium channel (Kv2.1); *KCNJ10*, a potassium Ca^2+^-activated channel (Kir4.1); and classified as Ca^2+^-binding protein; *PRKCG*, Ca^2+^-dependent serine/threonine protein kinase Cγ (PKCγ); *PRKCB*, Ca^2+^-dependent serine/threonine protein kinase Cβ (PKCβ); and *CAMK2A*, the Ca^2+^-calmodulin-dependent protein kinase IIα. KCNIP proteins are known to control gene transcription directly by binding to DRE sites. To test whether KCNIP proteins may be involved in the regulation of the expression of these selected prognosis genes, we searched for the presence of DRE sites within their proximal promoters. The *CACNA1H* and *PRKCB* genes present both one DRE potential site in their proximal promoter and *KCNB1* presents two DRE-binding sites (Table 3). More exciting are the three and four putative DRE sequences exhibited by *CAMK2A* and *KCNN4*, respectively, ideally positioned between the TATA box and the start codon, within the highly conserved sequence of proximal promoter in primates (Figure 1). The *CAMK2A* proximal promoter is also particularly conserved in mouse compared to human (87%), their DRE sites respect orientation and repartition, even for tandem organization. This promising observation has to be tested for KCNIP binding efficiency. Recent evidence argues for the existence of functional DRE sites within the *CAMK2A* proximal promoter. KCNIP3 mutants with two amino acids substitution in the EF-hands two, three, and four are unable to respond to Ca^2+^ and function as a constitutively dominant active (daDREAM) transcriptional repressor (Savignac et al., 2005). In transgenic mice with neuronal expression of this daDREAM, the *CAMK2A* mRNA level is reduced by 1.7-fold compared to wild type (Benedet et al., 2017). Mouse promoter for *KCNN4* is conserved (79%), but in a lesser extend concerning DRE sequences. These sequence alignments for proximal promoters let guess a putative regulatory role of KNCIPs in the expression of some prognosis genes in GBM. Of note, not all of these prognosis genes exhibit DRE-like sites, as no DRE putative sequence was detected for *KCNJ10* or *PRKCG* (Table 3), suggesting that KCNIPs are not the only transcriptional regulators directly implicated in the regulation of these prognosis genes, but the hypothesis of their contribution remains attractive. It is noteworthy that the previous results were obtained using transcriptomic data issued from DNA chips. When using the portal UALCAN (Chandrashekar et al., 2017) interfaced with the TCGA data base of transcriptomic cancer profiles obtained by RNA-seq techniques, only *CACNA1H* and *KCNN4* expression levels are correlated with significant differences in survival curves. It is noticeable that these two genes present one and three DRE sites, respectively. Anyhow, the presence of these putative DRE sites on prognosis genes, suggests that remodeling of Ca^2+^ homeostasis in GBM stem cells may contribute to the alteration of the expression of these prognosis genes. These preliminary observations urge for a more complete analysis taking into account the high level of false negatives when using the transcriptomic signatures built from DNA chip data.

**Table 3:** Candidate genes associated with unfavorable prognosis in GBM.

Gene	Function	Role in GBM	Reference	Number of putative DRE sites	Sense/position in bp^a^	Sequence in *Homo sapiens* (core sequence underlined)
*CACNA1H*	Cav3.2; T-type Ca^2+^ channel; Ca^2+^ homeostasis	Over-expression associated with worse prognosis	Zhang et al., 2017	1	AS/-72	5′tccgcgg**tgac**cgcgccg3′
*KCNN4*^b^	KCa3.1; voltage-independent potassium Ca^2+^-activated channel	Over-expression associated with worse prognosis, confers invasive phenotype.	Turner et al., 2014; Wang et al., 2016	4	S/-359 AS/-334 S/-166 AS/-94	5′ggtgtgt**gtca**caaagtac3′ 5′ttgtgtg**tgac**aaagccca3′ 5′cctggcc**gtca**ccactccc3′ 5′agcaggc**tgac**gacctgca3′
*KCNB1*	Kv2.1; potassium voltage-gated channel; delayed rectifier potassium channel	Downregulated in gliomas. Correlated with malignant progression when associated with KCNN4 and KCNJ10	Wang et al., 2016	2	AS/-137 AS/-48	5′acggccg**tgac**gcgcgccc3′ 5′cgtcgag**tgac**agcggcct3′
*KCNJ10*	Kir4.1; potassium voltage-gated channel; ATP-dependent inwardly rectifier; potassium buffering in glial cells	Downregulated in gliomas, correlated with malignant progression when associated with KCNN4 and KCNB1		NF		
*PRKCG*	Protein kinase Cγ; serine/threonine protein kinase activated by Ca^2+^ and diacylglycerol	Belong to a co-expression network genes that can serve as prognostic factors for GBM	Zhang et al., 2018	NF		
*PRKCB*	Protein kinase Cβ; serine/threonine protein kinase activated by Ca^2+^ and diacylglycerol			1	AS/-168	5′gggcgag**tgac**agccccgg3′
*CAMK2A*^C^	Ca^2+^-calmodulin-dependent protein kinase IIα			3	AS/-192 S/-133 S/-129	5′tggatgc**tgac**gaaggctc3′ 5′ggctc**gtcagtcaa**accgg3′


*^a^Position upstream from the ATG; S, sense; AS, anti-sense; ^*b*^putative DRE sites in close proximity; ^*c*^two putative DRE sites in tandem. NF, not found, in accordance with the criteria in proximal 5′ upstream sequence between the tata box and the start codon (see details in text).*

## Perspectives/Prospect on KCNIPs in GBM

Although no specific data are available for KCNIPs’ function in GBM or even cancers, one can speculate taking into account published functions of KCNIP in other cell types. KCNIPs are in fact multifunctional EF hand Ca^2+^-binding proteins and according to their interaction partners and subcellular localization one can discriminate at least three main functions: (1) regulation of cellular excitability, (2) regulation of intracellular signaling, and (3) control of transcription.

### Control of Cellular Excitability

The control of cellular excitability which involves the formation of a macromolecular signaling complex between KCNIP1 or 2, the A-type Kv4 potassium channel, and the T-type Ca^2+^ channel Cav3 (Anderson et al., 2010a,b) is unlikely to occur in GSLCs. Indeed, investigation of the electrophysiological properties of glioma cells revealed the absence of A-type potassium channels in these cells (Bordey and Sontheimer, 1998). Therefore, only the two other functions of KCNIP may be relevant to GBM physiology.

### Regulation of Intracellular Ca^2+^ Signaling

In cardiomyocytes, KCNIP2 participates in the modulation of Ca^2+^ release through ryanodine receptors (RyR) by interacting with the ryanodine modulator, presenilin (Nassal et al., 2017). The presenilin/KCNIP3 complex has also been shown to modulate IP3-mediated Ca^2+^ release (Leissring et al., 2000). We have already shown that the unique drug able to kill quiescent GSLCs acts through a modulation of IP3 signaling (Dong et al., 2017).

### Control of Transcription

As mentioned above, all KCNIPs can bind to DRE sites on DNA and directly control transcription. KCNIP3 (DREAM) can also interact with other transcription factors such as CREB and therefore affects transcription of genes that do not contain DRE sites (review in Rivas et al., 2011). Interestingly, in cardiomyocytes, it has been shown that the complex Ca^2+^/CAMK2 regulates nuclear translocation of KCNIP3 (Ronkainen et al., 2011). As CAMK2A has been identified as a prognosis gene in GBM (Table 3), such a mechanism is likely to occur in GBM.

In conclusion, since no experimental data exists for the moment in the literature concerning the function of KCNIP family in GBM, this opens a new field of research. In other models, KCNIPs have pleiotropic effects. Their well-known role as transcriptional repressors, and the presence of DRE sites in the promoter region of some GBM prognosis genes argue for a transcriptional function of KCNIPs in GBM. However, non-transcriptional roles have also to be considered more closely in the future.

## Author Contributions

IN, JH, M-CK, FA, MM, and CL designed the experiments. IN, FA, and CL performed and analyzed the experiments. IN, JH, M-CK, MM, and CL wrote the manuscript. JH, MM, and CL analyzed the data, provided financial support, and the final approval of manuscript. All authors reviewed the manuscript.

## Conflict of Interest Statement

The authors declare that the research was conducted in the absence of any commercial or financial relationships that could be construed as a potential conflict of interest.
